# Effects of Systemic Profiles on Choroidal Thickness in Treatment-Naïve Eyes With Diabetic Retinopathy

**DOI:** 10.1167/iovs.61.11.12

**Published:** 2020-09-09

**Authors:** Min Gyu Choi, Hum Chung, Young Hee Yoon, Jee Taek Kim

**Affiliations:** 1 Department of Ophthalmology, College of Medicine, Chung-Ang University Hospital, Seoul, South Korea; 2 510 Air Defense Artillery Battery, 1st Air Defense Missile Brigade, Air Defense & Guided Missile Command, Republic of Korea Air Force, Pohang, Korea; 3 The One Seoul Eye Clinic, Gangnamdaero 624, Gangnam-gu, Seoul, South Korea; 4 Department of Ophthalmology, Asan Medical Center, College of Medicine, University of Ulsan, Seoul, South Korea

**Keywords:** diabetic retinopathy, diabetic kidney disease, choroidal thickness, diabetic choroidopathy

## Abstract

**Purpose:**

The purpose of this study was to analyze the effects of systemic and ocular profiles on subfoveal choroidal thickness (SFChT) in treatment-naïve eyes with diabetic retinopathy (DR).

**Methods:**

This study included patients with treatment-naïve DR. They underwent routine laboratory evaluations, including complete blood cell count, liver function tests, kidney function tests, and urinalysis for macroalbuminuria. The systemic and ocular factors associated with the change in SFChT in DR were analyzed.

**Results:**

A total of 136 eyes from 136 patients with diabetes and 30 eyes from 30 age-matched healthy controls were recruited. Generalized linear model analyses showed that the SFChT in treatment-naïve eyes with DR was positively associated with the DR grade and estimated glomerular filtration rate (eGFR; *P* = 0.001) and negatively associated with age (*P* < 0.001) and serum phosphorus levels (*P* = 0.001). Treatment-naïve eyes with proliferative DR (PDR; 313.4 ± 9.0 µm) or severe nonproliferative DR (NPDR; 299.7 ± 9.7 µm) had thicker choroid than eyes with mild to moderate NPDR (251.7 ± 11.1 µm) or no DR (231.2 ± 14.5 µm) after adjusting for age, eGFR, and phosphorus levels.

**Conclusions:**

Choroid is affected by renal function and the grade of DR in patients with diabetes. Advanced retinopathy is associated with choroidal thickening, and the severity of concomitant renal disease is associated with choroidal thinning.

Diabetic retinopathy (DR) is the leading cause of vision loss and blindness in advanced and developing countries. This diabetic choroidopathy has been previously investigated by several studies using electron microscopic analysis, immunohistochemical analysis, ocular blood flow measurement, and indocyanine green angiography (ICGA).^1^^–^^4^

The advent of enhanced-depth imaging optical coherence tomography has facilitated intense investigation of the choroidal thickness in patients with DR. We previously reported that subfoveal choroidal thickness (SFChT) increases as the disease progresses (in severity) from severe nonproliferative DR (NPDR) to treatment-naïve (naïve) proliferative DR (PDR).^5^ However, this phenomenon was deemed controversial until now. Rewbury et al. have reported findings consistent with the abovementioned observation, demonstrating that the SFChT in the PDR group was higher than that in the mild NPDR group.^6^ On the contrary, several other studies have suggested that the SFChT decreases in patients with diabetes.^7^^–^^10^ The findings on the effects of diabetic macular edema (DME) on SFChT are also inconsistent, in addition to the controversy regarding the effect of DR on the choroid.^11^^,^^12^ A few studies have reported the systemic effects of microalbuminuria and chronic kidney disease (CKD) on the choroid; however, these findings are also inconsistent.^13^^–^^15^ Farias et al. reported a thinner choroid in the microalbuminuric group compared with the normoalbuminuric group.^13^ Furthermore, Malerbi et al. reported greater SFChT in the microalbuminuric group than that in the normoalbuminuric group.^14^ These inconsistencies in the state of the SFChT in patients with DR, DME, and microalbuminuria suggest the presence of several confounding factors, including the effects of DR severity, previous treatment, and systemic status. However, the association between the changes in SFChT and systemic profiles associated with CKD has not been systematically investigated until now.

Thus, this study was designed to contribute evidence to these ongoing debates. This study aimed to analyze the effects of systemic and ocular profiles on SFChT in treatment-naïve eyes with DR.

## Methods

### Participants

This retrospective observational cross-sectional study was approved by the Institutional Review Board of Chung-Ang University Hospital, Seoul, South Korea, and was conducted according to the tenets of the Declaration of Helsinki. The medical records of consecutive patients who visited both the diabetes and retina clinics of Chung-Ang University Hospital between September 1, 2016, and April 30, 2018, were retrospectively reviewed.

### Inclusion and Exclusion Criteria

Only treatment-naïve eyes without any previous ocular treatment for DR or DME were included. All patients underwent complete ophthalmologic examination, including the measurement of best-corrected visual acuity (BCVA), intraocular pressure (IOP), and refractive error; slit lamp examination; fundus examination and photography; and swept-source optical coherence tomography (SS-OCT). For regular systemic workup, the following examinations were performed: body weight, height, body mass index (BMI), blood pressure (BP), complete blood cell count, hemoglobin A1c (HbA1c) level, liver function tests, and kidney function tests. Microalbumin and creatinine levels in urine and the urinary (micro)albumin/creatinine ratio (ACR) were also analyzed. Only patients who underwent systemic workup within a span of 4 weeks of the ophthalmologic evaluations were selected.

The exclusion criteria included prior retinal surgery or panretinal photocoagulation (PRP), intravitreal injection, sub-Tenon's injection, history of ocular trauma or any other eye diseases (such as retinal and choroidal diseases), refractive error greater than ± 3.0 diopter (D), and systemic diseases besides diabetes or hypertension. Eyes with low-quality OCT images (image quality index < 60) or media opacities, such as vitreous hemorrhage, were also excluded.

### Data Collection

The severity of DR was graded according to the Early Treatment Diabetic Retinopathy Study (ETDRS) retinopathy severity scale as follows: no DR, mild to moderate or severe NPDR, and PDR. Fluorescein angiography was performed to grade the severity of DR using an ultra-wide-field confocal scanning laser ophthalmoscope (Optos Panoramic 200MA; Optos PLC, Dunfermline, Scotland, UK) in eyes with severe NPDR or PDR.

Furthermore, SS-OCT (DRI Triton; Topcon, Tokyo, Japan) was performed, with a wavelength of 1050 nm and a scan speed of 100,000 amplitude-scans/s, which yielded an axial resolution of 8 µm and a depth of 2.4 dB/mm. Optical coherence tomography (OCT) B-scan was performed with a 6 × 6-mm three-dimensional cube-scan and a 9-mm 5-line cross-scan. The central retinal thickness (CRT) was obtained from an automatic ETDRS grid map in the 6 × 6-mm 3-dimensional cube-scan mode after confirmation of the position of the grid. The SFChT was measured at the subfoveal region from the 9-mm 5-line cross-scan, using a built-in caliper tool, as the distance between Bruch's membrane and the choroid-sclera junction. Two independent observers (M.G.C. and J.T.K.), who were blinded to the clinical data of each patient, measured the SFChT, and the average value was used to avoid interobserver variation. Control data were obtained from the eyes of age-matched patients who visited the retina clinic for treatment of idiopathic epiretinal membrane or macular hole.

The following five ocular parameters from one eye (right eye) of each patient were recorded and analyzed to investigate the correlation with SFChT: BCVA, refractive error, IOP, CRT, and grade of DR. The following 23 systemic profiles were recorded to analyze the correlation between SFChT and the parameters obtained from the systemic workup: existence of hypertension, systolic BP, diastolic BP, age, BMI, hemoglobin, hematocrit, albumin, albumin/globulin ratio, total bilirubin, direct bilirubin, alanine transaminase, aspartate transaminase, alkaline phosphatase, γ-glutamyl transferase, total cholesterol, creatinine (Cr), blood urea nitrogen/creatinine ratio, estimated glomerular filtration rate (eGFR), calcium, phosphorus, urinary microalbumin level, and urinary ACR.

### Statistical Analyses

Statistical analyses were performed using the Statistical Package for the Social Sciences software (version 25.0; International Business Machines Corp., Armonk, NY, USA) using one-way analysis of variance and analysis of covariance with post hoc analysis. Simple linear regression analyses were used to analyze the ocular or systemic factors that affect the SFChT. Statistical significance was defined by a *P* value < 0.05. Additionally, generalized linear model was used to analyze the categorical and numerical parameters using the factors with *P <* 0.05 in simple linear regression. A total of 28 factors were analyzed for correlation with SFChT. Therefore, statistical significance was adjusted as a *P* value < 0.0017 (0.05/28) using Bonferroni correction for multiple testing.

## Results

### Baseline Characteristics

A total of 136 eyes with treatment-naïve DR from 136 patients with type 2 diabetes (69 men and 67 women, mean age: 54.8 ± 11.0 years) and 30 eyes from 30 age-matched controls were included in this study. The mean duration of diabetes was 13.7 ± 17.4 years. The eyes were grouped according to the severity of DR: no DR (30 eyes from 30 patients), mild to moderate NPDR (31 eyes from 31 patients), severe NPDR (38 eyes from 38 patients), and treatment-naïve PDR (37 eyes from 37 patients).

Age, BMI, existence of hypertension, duration of diabetes, and IOP did not differ significantly among the groups. However, systolic and diastolic BP, BCVA, refractive error, fasting blood sugar levels, and HbA1c levels were (statistically) significantly different between the groups. The baseline characteristics of the patients in each group are presented in Table 1.

**Table 1.** Baseline Characteristics of Patients

	Healthy Control	No DR	Mild to Moderate NPDR	Severe NPDR	Treatment-Naïve PDR	*P* Value^*^
Number of patients	30	30	31	38	37	–
Number of eyes	30	30	31	38	37	–
Number of eyes of HTN, *n* (%)	0 (0)	4 (40.0)	36 (65.5)	44 (65.7)	40 (66.7)	–
SBP, mm Hg	125.2 ± 9.2	123.0 ± 12.8	122.9 ± 12.8	130.1 ± 20.1	131.9 ± 23.1	0.027
DBP, mm Hg	70.3 ± 5.5	70.2 ± 6.6	70.7 ± 8.6	75.4 ± 12.4	82.1 ± 17.3	<0.001
BMI, kg/m^2^	25.2 ± 2.8	24.4 ± 4.6	26.3 ± 3.5	25.2 ± 3.4	25.8 ± 4.7	0.347
Age, y	56.8 ± 14.0	58.5 ± 13.2	58.5 ± 11.1	56.5 ± 9.1	52.3 ± 10.0	0.264
BCVA, logMAR	0.06 ± 0.08	0.08 ± 0.06	0.07 ± 0.09	0.10 ± 0.20	0.21 ± 0.32	0.012
Refractive error, spherical equivalent	−0.42 ± 1.82	0.51 ± 0.42	−0.59 ± 1.57	−0.68 ± 1.99	−1.34 ± 1.83	0.012
Intraocular pressure, mm Hg	15.9 ± 3.0	15.7 ± 4.5	16.4 ± 3.3	15.8 ± 2.9	15.9 ± 2.8	0.877
CRT in all eyes, µm	221.0 ± 22.3	231.4 ± 23.6	246.4 ± 29.3	241.3 ± 49.8	256.5 ± 58.4	0.006
DM duration, y		8.1 ± 5.0	13.1 ± 8.6	17.4 ± 27.8	11.0 ± 7.5	0.146
FBG, mg/dL		126.4 ± 20.5	146.8 ± 59.1	157.5 ± 59.5	187.4 ± 134.4	0.002
HbA1c, %		9.0 ± 3.1	7.9 ± 0.6	7.7 ± 1.4	8.8 ± 2.3	0.032

Data are presented as number (%) or mean ± standard deviation, unless otherwise indicated.

BMI, body mass index; BCVA, best-corrected visual acuity; CRT, central retinal thickness; DBP, diastolic blood pressure; DM, diabetes mellitus, DR, diabetic retinopathy; FBG, fasting blood glucose; HbA1c, hemoglobin A1c; ΔHbA1c, difference in HbA1c levels between the present and previous examinations; HTN, hypertension; logMAR, Logarithm of the Minimum Angle of Resolution; NPDR, nonproliferative diabetic retinopathy; PDR, proliferative diabetic retinopathy; SBP, systolic blood pressure.

* Analysis of variance among the groups.

Patients with severe NPDR or PDR had a tendency toward advanced severity with respect to glucose control, hypercholesterolemia, and CKD. The following parameters were also statistically significantly different among the groups: albumin/globulin ratio and albumin, direct bilirubin, total cholesterol levels, Cr levels, eGFR, urinary microalbumin levels, and urinary ACR. The changes in the systemic profiles of the patients in each group are presented in Table 2. Additionally, the distribution of patients according to the severity of DR and eGFR is shown in Supplementary Table S1.

**Table 2.** Comparison of Blood and Urine Profile in Patients According to Diabetic Retinopathy

	No DR	Mild to Moderate NPDR	Severe NPDR	Treatment-Naïve PDR	*P* Value^*^
Hemoglobin, g/dL	13.8 ± 1.0	18.7 ± 25.3	17.1 ± 22.3	12.7 ± 2.3	0.365
Hematocrit, %	39.5 ± 3.3	41.0 ± 4.0	39.4 ± 5.1	38.7 ± 6.0	0.074
Total protein, g/dL	7.0 ± 0.2	7.1 ± 0.4	7.0 ± 0.3	6.9 ± 0.7	0.374
Albumin, g/dL	4.2 ± 0.4	4.2 ± 0.3	4.2 ± 0.4	3.9 ± 0.5	0.002^†^
Albumin/ globulin ratio	0.60 ± 0.04	0.60 ± 0.04	0.60 ± 0.05	0.56 ± 0.04	<0.001^†^
Total bilirubin, mg/dL	0.72 ± 0.28	0.64 ± 0.24	0.58 ± 0.25	0.55 ± 0.23	0.089
Direct bilirubin, mg/dL	0.22 ± 0.08	0.21 ± 0.1	0.18 ± 0.11	0.16 ± 0.09	0.042^†^
AST, IU/L	23.6 ± 6.8	22.1 ± 11.9	24.0 ± 9.0	22.5 ± 8.1	0.685
ALT, IU/L	19.2 ± 8.2	18.3 ± 10.7	23.9 ± 13.2	21.0 ± 11.3	0.096
ALP, IU/L	76.4 ± 11.7	80.1 ± 26.4	77.6 ± 30.2	86.9 ± 29.8	0.284
GGT, IU/L	22.8 ± 5.3	28.8 ± 41.2	28.4 ± 22.4	38.8 ± 33.4	0.182
Total cholesterol, mg/dL	161.4 ± 31.1	144.1 ± 29.8	142.5 ± 38.8	186.0 ± 67.2	<0.001^†^
BUN/Cr ratio	19.0 ± 6.1	21.9 ± 6.1	19.8 ± 7.7	19.7 ± 6.9	0.261
Creatinine, mg/dL	0.89 ± 0.24	0.81 ± 0.25	0.99 ± 0.52	1.17 ± 0.91	0.01^†^
eGFR, mL/min/1.73 m^2^	86.4 ± 7.7	97.2 ± 22.7	86.7 ± 32.2	81.4 ± 34.1	0.026^†^
Calcium, mg/dL	9.3 ± 0.3	9.1 ± 0.3	9.1 ± 0.3	9.0 ± 0.4	0.051
Phosphorus, mg/dL	3.2 ± 0.2	3.8 ± 0.6	3.7 ± 0.5	3.8 ± 0.6	0.066
Urine microalbumin, mg/L	98.4 ± 87.8	144.2 ± 317.8	434.8 ± 1509.5	1631.0 ± 2981.5	<0.001
ACR, µg/mg Cr	63.3 ± 120.2	167.7 ± 500.5	280.4 ± 610.8	1189.9 ± 1827.3	<0.001

Data are presented as mean ± standard deviation, unless otherwise indicated.

ACR, albumin/creatinine ratio of urine; ALP, alkaline phosphatase; ALT, alanine transaminase; AST, aspartate transaminase; BUN, blood urea nitrogen; Cr, creatinine; DR, diabetic retinopathy; eGFR, estimated glomerular filtration rate; GGT, γ-glutamyl transferase; NPDR, nonproliferative diabetic retinopathy; PDR, proliferative diabetic retinopathy.

* Analysis of variance among the groups.

† *P* < 0.05, post hoc analyses are not shown.

### Association Between Subfoveal Choroidal Thickness and Systemic Factors Related with Diabetes

**Figure 1.**
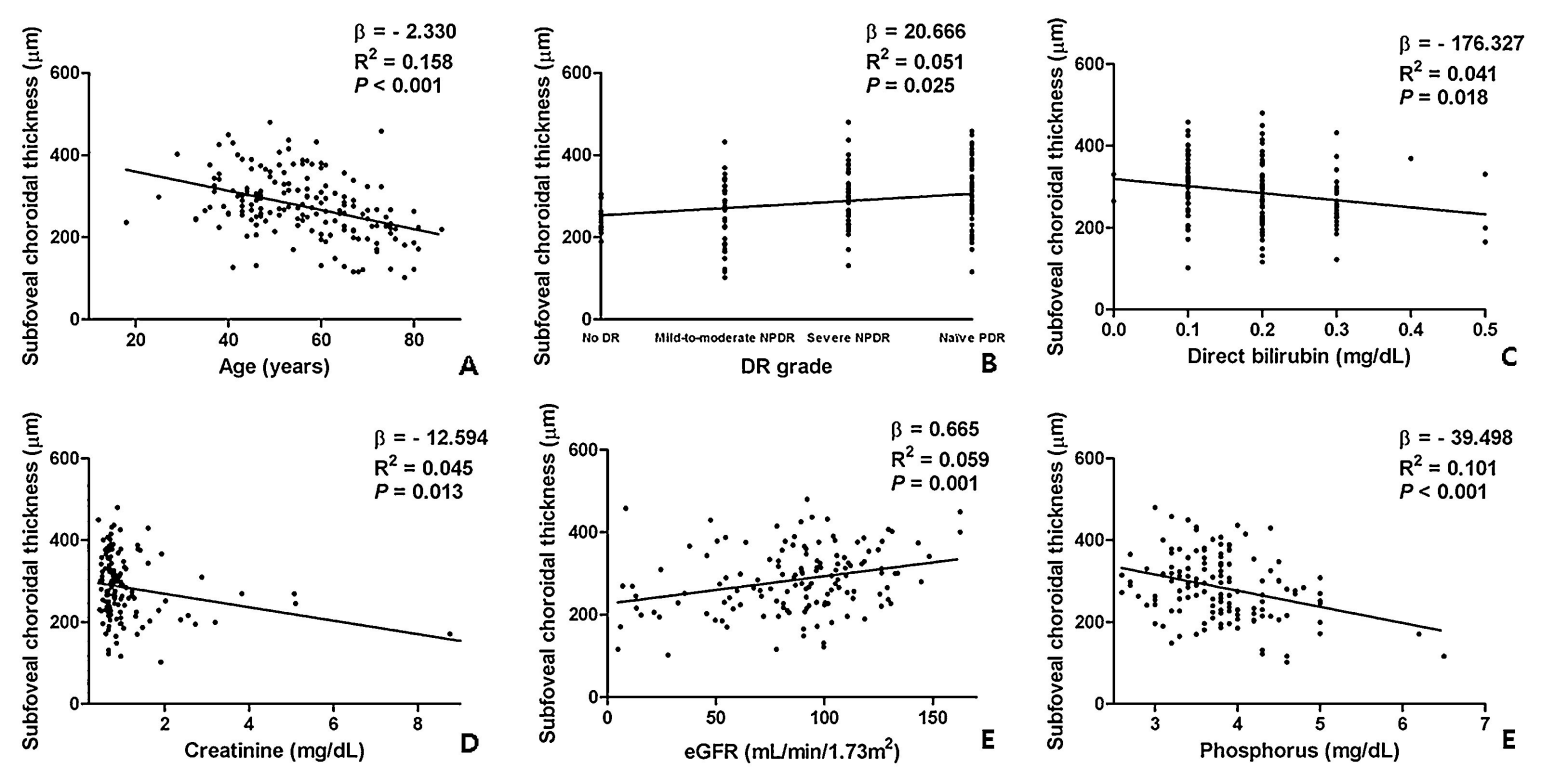
Scatter plot with simple linear regression assessing the association between subfoveal choroidal thickness (SFChT) and a few ocular and systemic parameters (*P* < 0.05), (**A, C, D,** **E**) Age and serum levels of direct bilirubin, creatinine, and phosphorus were negatively correlated with SFChT. (**B, E**) The grade of diabetic retinopathy and estimated glomerular filtration rate were positively correlated with SFChT.

**Figure 2.**
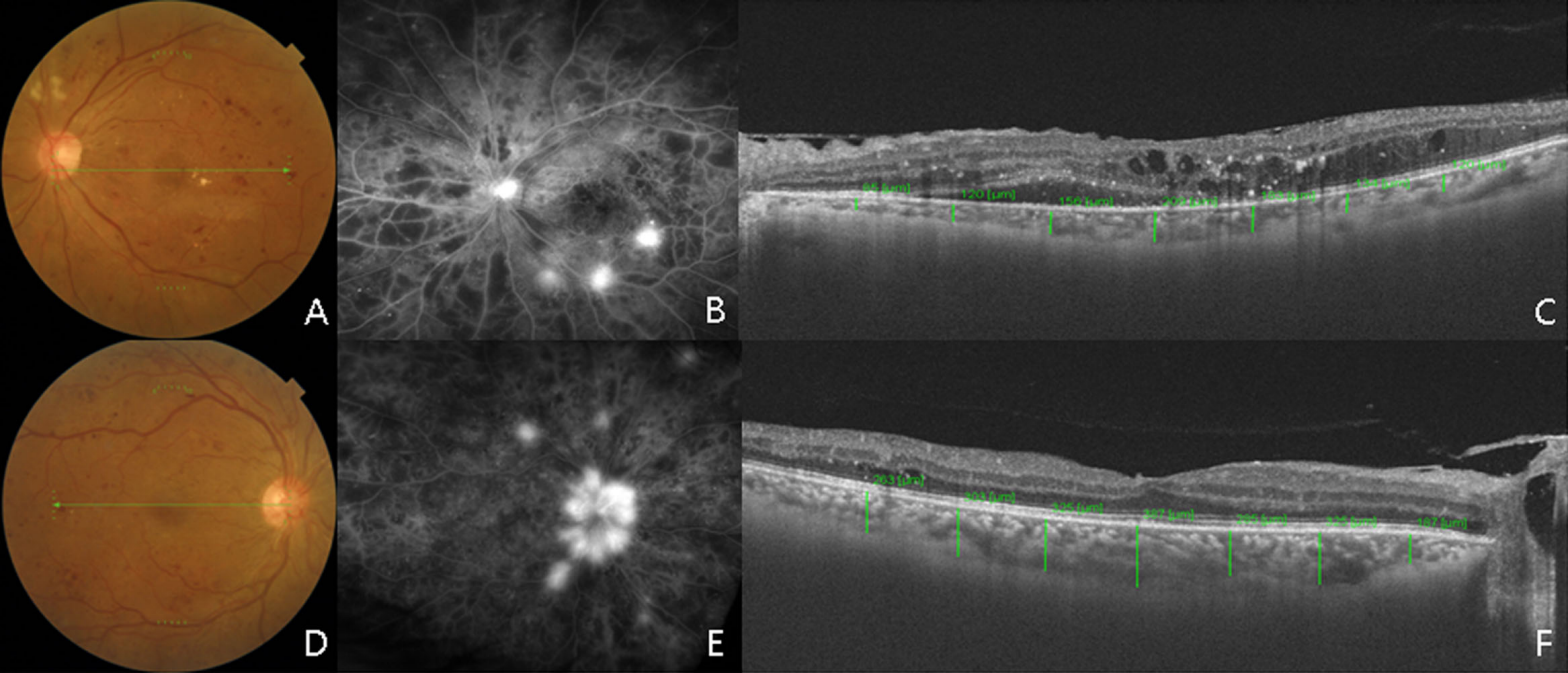
Two representative cases showing choroidal thinning and thickening in treatment-naïve eyes with proliferative diabetic retinopathy (PDR). Patients with choroidal thinning were in a more advanced stage of chronic kidney disease (CKD) than those with choroidal thickening. (**A**) Representative cases of choroid thinning (subfoveal choroidal thickness, 209 µm) in the eyes (with treatment-naïve PDR) of a 47-year-old woman with advanced CKD. Her ocular and systemic parameters were as follows: BCVA, 20/40; spherical equivalent, −1.0; hemoglobin A1c, 10.9%; fasting blood glucose (FBG), 135 mg/dL; Cr, 3.2 mg/dL; eGFR, 15.53 mL/min/1.73 m^2^, and phosphorus, 5 mg/dL. (**B**) Representative case of choroidal thickening (subfoveal choroidal thickness, 387 µm) in the eyes (with treatment-naïve PDR) of a 45-year-old man without CKD. His ocular and systemic parameters were as follows: BCVA, 20/25; spherical equivalent, −2.25; hemoglobin A1c, 9.7%; FBG, 329 mg/dL; Cr, 0.71 mg/dL; eGFR, 131.17 mL/min/1.73 m^2^, and phosphorus, 3.7 mg/dL. BCVA = best-corrected visual acuity; Cr, creatinine; CKD, chronic kidney disease; eGFR, estimated glomerular filtration rate; FBG, fasting blood glucose.

Simple linear regression analysis revealed that a greater SFChT was significantly associated with the following parameters: younger age (*P <* 0.001), advanced grade of DR (*P* = 0.025), lower levels of direct bilirubin (*P* = 0.018), Cr (*P* = 0.013) and phosphorus levels (*P* < 0.001), and higher levels of eGFR (*P* = 0.001; Figure 1, Table 3). Generalized linear model analysis revealed that a greater SFChT was associated with younger age (*P* < 0.001), advanced grade of DR, higher eGFR (*P* = 0.001), and lower phosphorus levels (*P* = 0.001; Table 4). Two representative cases of choroidal thinning and thickening in the treatment-naïve eyes with PDR in patients with and without CKD are shown in Figure 2.

**Table 3.** Univariate Analyses of the Association Between Subfoveal Choroidal Thickness and Systemic and Ocular Parameters

	Univariate Linear Regression		
	Regression Coefficient	*R* *^2^*	*P* Value
Age, y	−2.330	0.158	<0.001
DR grade	20.666	0.051	0.025
Direct bilirubin	−176.327	0.041	0.018
Creatinine, mg/dL	−12.594	0.045	0.013
eGFR, mL/min/1.73 m^2^	0.665	0.059	0.001
Phosphorus, mg/dL	−39.498	0.101	<0.001

DR, diabetic retinopathy; eGFR, estimated glomerular filtration rate.

**Table 4.** Generalized Linear Model for the Association Between Subfoveal Choroidal Thickness and Systemic and Ocular Parameters

Variables	*B* ^*^	SE	*P* Value
DR Grade			
No DR	0^†^	–	
Mild to moderate NPDR	14.548	17.9437	0.418
Severe NPDR	53.765	17.7145	0.002
Naïve PDR	65.806	17.9275	<0.001
Age, y	−1.995	0.438	<0.001
eGFR, mL/min/1.73 m^2^	0.675	0.158	0.001
Direct bilirubin	−99.441	60.3388	0.099
Cr	3.945	6.0492	0.514
Phosphorus, mg/dL	−33.505	8.622	0.001

Cr, creatinine; DR, diabetic retinopathy; eGFR, estimated glomerular filtration rate; NPDR, nonproliferative diabetic retinopathy; PDR, proliferative diabetic retinopathy.

* Unstandardized (B) coefficient.

† Set to zero because this parameter is redundant.

### Changes in SFChT in Eyes with Diabetic Retinopathy

**Figure 3.**
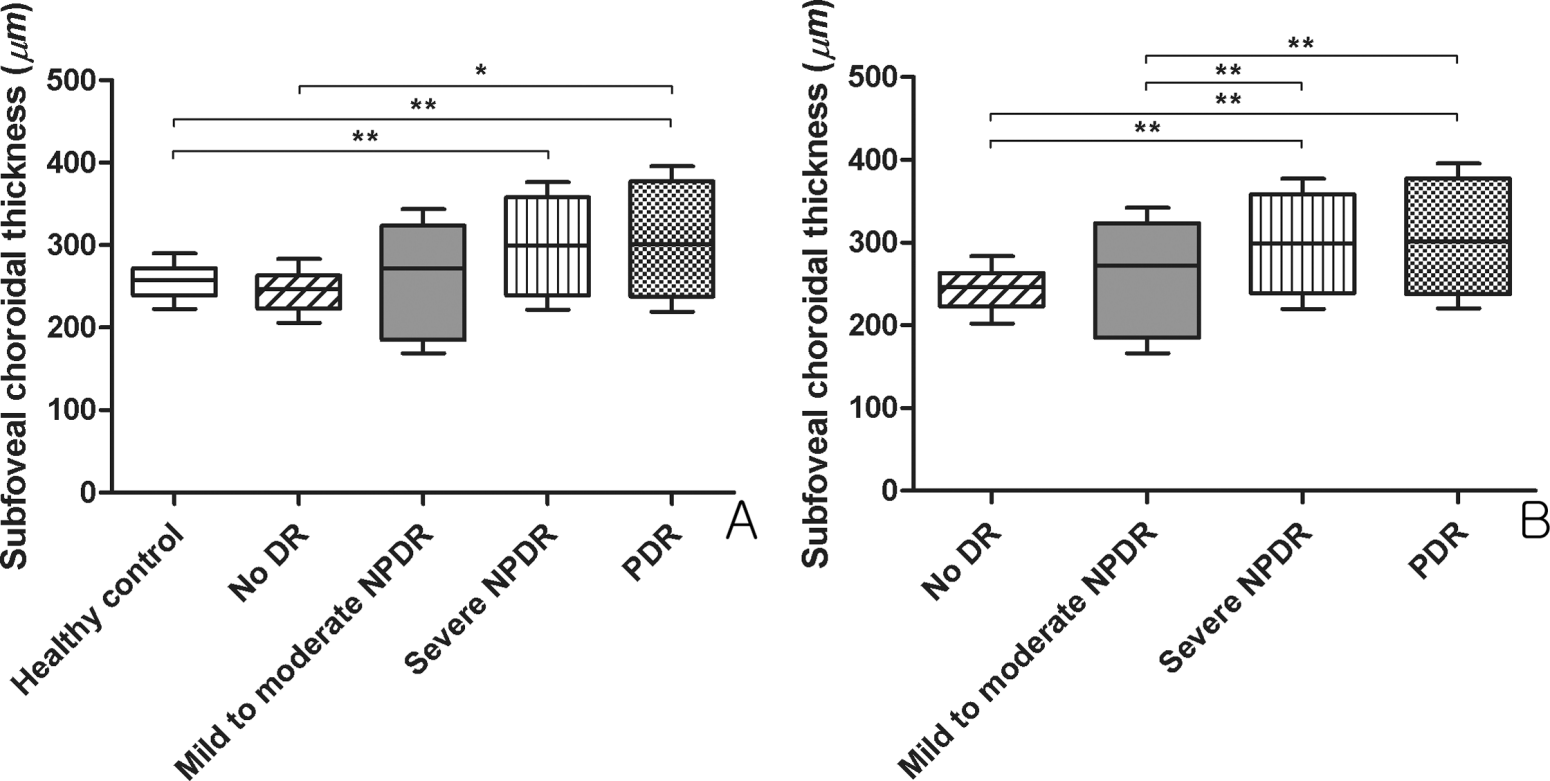
Changes in subfoveal choroidal thickness (SFChT) based on the severity of diabetic retinopathy (DR) (**A**) The changes of SFChT according to DR stage after age adjustment. The eyes with treatment-naïve PDR and severe NPDR had greater SFChT than the eyes of healthy controls and no DR. (**B**) Changes in SFChT according to the stage of DR after adjusting for age, phosphorus levels, and estimated glomerular filtration rate. Eyes with treatment-naïve PDR and severe NPDR had greater SFChT than eyes with mild to moderate NPDR and no DR. Analysis of covariance with post hoc analysis: ^*^P < 0.05; ^**^P < 0.01.

The interobserver reproducibility of SFChT measurement ranged from 0.985 to 0.991. Analysis of covariance with post hoc analysis revealed that the age-adjusted SFChT in treatment-naïve eyes with PDR (299.4 ± 9.6 µm) or severe NPDR (299.7 ± 10.8 µm) was greater than that in normal controls (272.6 ± 11.4 µm) or no DR (250.2 ± 15.7 µm). The SFChT in treatment-naïve eyes with PDR (313.4 ± 9.0 µm) or severe NPDR (299.7 ± 9.7 µm) was greater than that of eyes with mild to moderate NPDR (251.7 ± 11.1 µm) or no DR (231.2 ± 14.5 µm) after adjusting for age, eGFR, and phosphorus levels. The DR-associated changes in choroidal thickness (ChT) are shown in Figure 3.

## Discussion

This study included a relatively large number of treatment-naïve eyes with severe NPDR and PDR to systematically investigate the effects of ocular and systemic factors on SFChT. It revealed that the SFChT in eyes with DR was affected by several systemic profiles associated with CKD and the severity of DR.

First, the grade of DR was found to be positively associated with the SFChT. Several studies have reported inconsistent findings, that is, they found that SFChT increased or decreased in advanced DR or DME.^5^^–^^12^ It is thought that these inconsistent findings may be attributed to several confounding factors. Thus, it is important to identify and control for such confounding factors. We have also shown that SFChT decreases in eyes treated with PRP.^5^ Recently, several studies have demonstrated that SFChT is affected by PRP.^16^^,^^17^ Therefore, it is thought that PRP treatment is the most important confounding factor, with respect to SFChT in patients with DR. PRP treatment was not considered in several previous studies that suggested decreased ChT in eyes with PDR.^7^^,^^8^^,^^18^ Thus, treatment-naïve PDR and PRP-treated PDR should be considered as separate categories while considering the effect of PRP treatment on the choroid. This study enrolled only those patients with treatment-naïve eyes to limit the number of variables, and found that SFChT was positively associated with the grade of DR. Moreover, the SFChT in treatment-naïve eyes with PDR and severe NPDR was greater than that in eyes with mild to moderate NPDR and no DR. These findings are consistent with those of our previous study that uses enhanced-depth imaging OCT.^5^

Several studies also have shown that the SFChT decreased after intravitreal injection of anti-vascular endothelial growth factor (VEGF), which suggests that the choroid is sensitive to intraocular VEGF concentration.^19^^,^^20^ The most important finding of the present study was the increase in the SFChT in eyes with treatment-naïve PDR, which had considerably high concentrations of VEGF.^21^^,^^22^ The choroidal thickening in treatment-naïve PDR might reflect the increased concentration of VEGF or a large area of nonperfusion. Therefore, the increase in the SFChT in treatment-naïve eyes with PDR may be suggestive of choroidal edema.

Second, eGFR and serum phosphorus levels (among the several systemic profiles) were found to be associated with SFChT. Interestingly, eGFR has been used widely to evaluate the kidney function in patients with CKD along with microalbuminuria.^23^ Balmforth et al. reported that choroidal thinning in patients with CKD was associated with lower eGFR.^15^ Mule et al. also described a correlation between ChT and renal function in patients with hypertension.^24^^,^^25^ The findings of the present study are consistent with those of previous studies, although the correlation between ChT and renal function has rarely been described in patients with diabetes or DR. Abnormality in mineral metabolism occurs from the early stage of CKD.^26^ Phosphorus is mainly excreted by the kidneys in normal conditions.^27^ The decrease in the infiltration and excretion of phosphorus with the progression of CKD leads to hyperphosphatemia, which is associated with the progression of secondary hyperparathyroidism.^26^^,^^28^^,^^29^ Furthermore, these changes are associated with the fragility and high turnover of bone, vascular calcification, and cardiovascular disease. Thus, hyperphosphatemia is considered to be an important marker of cardiovascular morbidity and mortality in patients with CKD.^28^^,^^30^ Interestingly, higher phosphorus levels that are within normal range are significantly associated with coronary infarction or coronary death even in patients with normal kidney function.^31^ Therefore, we believe that choroidal thinning in naïve eyes with severe NPDR or PDR can suggest or predict vascular calcifications of the cardiovascular system.

Third, no correlation was observed between SFChT and microalbuminuria or ACR in the present study. Microalbuminuria, ACR, and eGFR are used as markers of renal damage.^23^ A few previous studies have reported a correlation between SFChT and microalbuminuria.^13^^,^^14^ Farias et al. reported a significantly thinner choroid in patients with microalbuminuria.^13^ On the contrary, Malebi et al. reported an increase in SFChT in patients with microalbuminuria.^14^ These inconsistent findings might also be attributed to several confounding factors, including variability in the grade of DR and the systemic profiles. Moreover, compensation of renal function after medication may also contribute to this inconsistency. Angiotensin-converting enzyme inhibitors or angiotensin receptor blockers ameliorate microalbuminuria and reduce the risk of progression to macroalbuminuria in patients with diabetes.^32^ Thus, the use of angiotensin-converting enzyme inhibitors and angiotensin receptor blockers is recommended in patients with CKD.^33^ The lack of an association between microalbuminuria and SFChT in the present study might be related to the effect of medications.

We hypothesized that increased level of VEGF from the nonperfusion area might lead to choroidal edema or choroidal vasodilation in eyes with severe NPDR or naïve PDR in patients without hyperphosphatemia, after considering the results of the present study. However, vascular calcification of the circulatory system might not only limit blood supply and choroidal thickening or edema but also lead to shrinkage or thinning of the choroid even in eyes with treatment-naïve PDR in patients with phosphorus retention. Specifically, in cases of both advanced retinopathy and concomitant nephropathy, we think that vascular calcification by advanced nephropathy might limit choroidal edema caused by advanced retinopathy and lead to choroidal shrinkage. Thus, we believe that the choroid was affected primarily by the systemic status and secondarily by the ocular status. This hypothesis partly explains both the thickening and thinning of the choroid in eyes with DR.

Only few earlier studies have investigated the findings of ICGA in eyes with DR.^3^^,^^34^ Weinberger et al. have described that 48% of eyes with NPDR showed diffuse late-phase hyperfluorescence on ICGA, corresponding to the area of capillary nonperfusion on fluorescein angiography and retinal edema.^3^ Moreover, 12% of eyes with NPDR showed spotty lobular hyper- and hypofluorescent areas. Shiragami et al. have described the three types of angiographic changes in eyes with DR as hypofluorescent spots in 80%, small hyperfluorescent spots in 68%, and large hyperfluorescent spots in 36% of eyes.^34^ We think that eyes with choroidal thickening may correspond to diffuse or large hyperfluorescent areas and a leaky choroid, and eyes with choroidal thinning may correspond to hyperfluorescent spots and choroidal atrophy. It would be interesting to investigate choroidal ischemia using both ICGA and OCT.

This study has several limitations. First, this was a retrospective observational study, and not a prospective one. Nevertheless, several confounding factors, including PRP and intravitreal injection, were controlled for in this study. Second, the enrolled participants were not medical treatment-naïve, although the eyes included were ophthalmologically treatment-naïve. Patients with DR were generally referred from other departments, including the endocrinology or nephrology department, after prescription of medication. Therefore, medical treatment-naïve patients were extremely rare among the patients who visited the retina clinic. Third, the interval between ophthalmologic examination and systemic workup was approximately 4 weeks, which might affect the investigated associations. Fourth, we did not evaluate the effects of DME. Considering the systemic effect on SFChT, we should compare the SFChT in eyes with unilateral DME and contralateral eyes without DME. However, the number of patients with unilateral DME was significantly small. Moreover, we included one eye per patient. Thus, the effect of DME on SFChT was not evaluated in this study. Fifth, from this study, we hypothesized that SFChT is more correlated with the ischemic area than DR severity. The nonperfusion areas were not measured in this study. Future studies using measurement of ischemic area and SFChT might support this hypothesis. Finally, not all changes in SFChT in eyes with DR can be explained by variations in the variables examined. A wide variety of confounding factors related to the patient's systemic health can affect the choroidal thickness.

In summary, we found a negative correlation between SFChT and a few systemic profiles associated with CKD and a positive correlation between SFChT and the grade of DR. We believe that this elucidation of the correlation between SFChT and systemic profiles in patients with DR might resolve the ongoing debate regarding the change in ChT in eyes with DR or DME. However, further prospective or longitudinal follow-up studies should be conducted to validate this conclusion.

## Supplementary Material

Supplement 1
