# Supplementary material for: Effects of Systemic Profiles on Choroidal Thickness in Treatment-Naïve Eyes With Diabetic Retinopathy
Source: Invest Ophthalmol Vis Sci. 2020 Sep 9;61(11):12. doi: 10.1167/iovs.61.11.12 (PMC7488646; doi:10.1167/iovs.61.11.12)
Supplement: Supplement 1 [file iovs-61-11-12_s001.pdf]

Table S1. Distribution of the included patients according to severity of diabetic retinopathy and chronic kidney disease.

Classification	No DR	Mild to moderate NPDR	Severe NPDR	Treatment-naïve PDR
eGFR≥90 mL/min/1.73 m^2	20 (66.7%)	19 (61.29%)	19 (50%)	18 (48.7%)
60≤eGFR<90 mL/min/1.73 m^2	7 (23.3%)	10 (32.3%)	8 (21.1%)	6 (16.2%)
30≤eGFR<60 mL/min/1.73 m^2	3 (10%)	1 (6.5%)	6 (18.8%)	5 (13.5%)
eGFR<30 mL/min/1.73 m^2	0 (0%)	1 (3.2%)	5 (13.2%)	8 (21.6%)
Total	30 (100%)	31 (100%)	38 (100%)	37 (100%)

Data are shown in number of the patients (percentage).
